# Supplementary material for: Multi‐dataset identification of innovative feature genes and molecular mechanisms in keratoconus
Source: J Cell Mol Med. 2024 Sep 19;28(18):e70079. doi: 10.1111/jcmm.70079 (PMC11412914; doi:10.1111/jcmm.70079)
Supplement: Supplementary file 2 — Material S2. [file JCMM-28-e70079-s002.zip › 7.GSEA analysis/6.SNORA74B/KEGG_CELL_ADHESION_MOLECULES_CAMS.html]

Details for gene set KEGG\_CELL\_ADHESION\_MOLECULES\_CAMS[GSEA]

|  || Dataset | SNORA74B.group.cls#h\_versus\_l |
| Phenotype | group.cls#h\_versus\_l |
| Upregulated in class | l |
| GeneSet | KEGG\_CELL\_ADHESION\_MOLECULES\_CAMS |
| Enrichment Score (ES) | -0.47019723 |
| Normalized Enrichment Score (NES) | -1.5072415 |
| Nominal p-value | 0.017077798 |
| FDR q-value | 0.31278056 |
| FWER p-Value | 0.768 |
Table: GSEA Results Summary

  

Fig 1: Enrichment plot: KEGG\_CELL\_ADHESION\_MOLECULES\_CAMS      
 Profile of the Running ES Score & Positions of GeneSet Members on the Rank Ordered List

  

| SYMBOL | TITLE | RANK IN GENE LIST | RANK METRIC SCORE | RUNNING ES | CORE ENRICHMENT || 1 | CLDN22 | na | 192 | 0.783 | 0.0274 | No |
| 2 | CD86 | na | 436 | 0.628 | 0.0468 | No |
| 3 | CLDN5 | na | 1859 | 0.384 | 0.0219 | No |
| 4 | SELP | na | 4012 | 0.260 | -0.0294 | No |
| 5 | CD22 | na | 4365 | 0.247 | -0.0291 | No |
| 6 | OCLN | na | 6320 | 0.182 | -0.0779 | No |
| 7 | ICOSLG | na | 6672 | 0.171 | -0.0809 | No |
| 8 | SELPLG | na | 6903 | 0.165 | -0.0806 | No |
| 9 | ITGA4 | na | 7435 | 0.150 | -0.0896 | No |
| 10 | CLDN23 | na | 7699 | 0.143 | -0.0912 | No |
| 11 | ITGB7 | na | 7862 | 0.139 | -0.0900 | No |
| 12 | MPZ | na | 7888 | 0.139 | -0.0849 | No |
| 13 | ITGAL | na | 7931 | 0.138 | -0.0803 | No |
| 14 | CD4 | na | 8358 | 0.128 | -0.0872 | No |
| 15 | CLDN20 | na | 8410 | 0.127 | -0.0834 | No |
| 16 | CNTN1 | na | 8657 | 0.121 | -0.0854 | No |
| 17 | CLDN7 | na | 8863 | 0.117 | -0.0864 | No |
| 18 | PDCD1LG2 | na | 8948 | 0.115 | -0.0840 | No |
| 19 | ITGA9 | na | 9643 | 0.100 | -0.0998 | No |
| 20 | SDC2 | na | 9903 | 0.095 | -0.1034 | No |
| 21 | NLGN3 | na | 10239 | 0.088 | -0.1093 | No |
| 22 | PDCD1 | na | 11152 | 0.072 | -0.1326 | No |
| 23 | CD28 | na | 11178 | 0.072 | -0.1303 | No |
| 24 | HLA-DRB5 | na | 11281 | 0.070 | -0.1303 | No |
| 25 | CLDN3 | na | 11534 | 0.066 | -0.1348 | No |
| 26 | CNTN2 | na | 11975 | 0.060 | -0.1450 | No |
| 27 | NLGN4X | na | 12158 | 0.058 | -0.1478 | No |
| 28 | CD99 | na | 12314 | 0.056 | -0.1500 | No |
| 29 | PTPRF | na | 12367 | 0.055 | -0.1492 | No |
| 30 | ICAM3 | na | 12456 | 0.054 | -0.1494 | No |
| 31 | ICAM2 | na | 12494 | 0.053 | -0.1483 | No |
| 32 | CLDN19 | na | 12660 | 0.051 | -0.1509 | No |
| 33 | CLDN16 | na | 12743 | 0.050 | -0.1511 | No |
| 34 | CD2 | na | 12955 | 0.047 | -0.1553 | No |
| 35 | HLA-F | na | 13085 | 0.046 | -0.1570 | No |
| 36 | CLDN8 | na | 13125 | 0.045 | -0.1563 | No |
| 37 | HLA-DQB1 | na | 13160 | 0.045 | -0.1553 | No |
| 38 | HLA-B | na | 13306 | 0.044 | -0.1577 | No |
| 39 | NEGR1 | na | 13420 | 0.042 | -0.1592 | No |
| 40 | CTLA4 | na | 13869 | 0.037 | -0.1705 | No |
| 41 | SELE | na | 14242 | 0.033 | -0.1799 | No |
| 42 | CLDN9 | na | 14622 | 0.029 | -0.1896 | No |
| 43 | MPZL1 | na | 14752 | 0.027 | -0.1922 | No |
| 44 | CDH1 | na | 15137 | 0.023 | -0.2024 | No |
| 45 | SDC1 | na | 15561 | 0.019 | -0.2138 | No |
| 46 | HLA-E | na | 15707 | 0.018 | -0.2172 | No |
| 47 | HLA-DOA | na | 16250 | 0.012 | -0.2324 | No |
| 48 | CLDN4 | na | 16601 | 0.008 | -0.2422 | No |
| 49 | F11R | na | 17230 | 0.002 | -0.2602 | No |
| 50 | ICOS | na | 17341 | 0.001 | -0.2633 | No |
| 51 | ALCAM | na | 17403 | 0.001 | -0.2651 | No |
| 52 | ITGB2 | na | 17416 | 0.000 | -0.2654 | No |
| 53 | HLA-C | na | 17481 | -0.000 | -0.2672 | No |
| 54 | SDC3 | na | 18016 | -0.006 | -0.2824 | No |
| 55 | PTPRC | na | 18235 | -0.009 | -0.2884 | No |
| 56 | HLA-A | na | 18401 | -0.011 | -0.2927 | No |
| 57 | HLA-G | na | 18916 | -0.016 | -0.3069 | No |
| 58 | HLA-DRB1 | na | 19037 | -0.017 | -0.3096 | No |
| 59 | HLA-DMA | na | 19878 | -0.027 | -0.3327 | No |
| 60 | SDC4 | na | 20085 | -0.029 | -0.3375 | No |
| 61 | CADM1 | na | 20334 | -0.032 | -0.3433 | No |
| 62 | CDH5 | na | 20522 | -0.034 | -0.3472 | No |
| 63 | CNTNAP1 | na | 20886 | -0.039 | -0.3561 | No |
| 64 | VCAN | na | 20971 | -0.040 | -0.3569 | No |
| 65 | NLGN2 | na | 21234 | -0.043 | -0.3626 | No |
| 66 | MAG | na | 21414 | -0.045 | -0.3659 | No |
| 67 | VCAM1 | na | 21476 | -0.046 | -0.3658 | No |
| 68 | NLGN1 | na | 21780 | -0.049 | -0.3724 | No |
| 69 | CD8B | na | 21807 | -0.049 | -0.3711 | No |
| 70 | CD276 | na | 22117 | -0.053 | -0.3778 | No |
| 71 | GLG1 | na | 22510 | -0.058 | -0.3867 | No |
| 72 | PECAM1 | na | 22774 | -0.062 | -0.3917 | No |
| 73 | CLDN15 | na | 22839 | -0.063 | -0.3909 | No |
| 74 | CLDN1 | na | 23337 | -0.070 | -0.4023 | No |
| 75 | HLA-DOB | na | 23582 | -0.074 | -0.4062 | No |
| 76 | CD58 | na | 23602 | -0.075 | -0.4036 | No |
| 77 | HLA-DRB4 | na | 24359 | -0.087 | -0.4218 | No |
| 78 | HLA-DRB3 | na | 24377 | -0.087 | -0.4187 | No |
| 79 | NEO1 | na | 24783 | -0.094 | -0.4264 | No |
| 80 | NRXN2 | na | 24842 | -0.096 | -0.4241 | No |
| 81 | L1CAM | na | 26099 | -0.119 | -0.4554 | No |
| 82 | CD8A | na | 26376 | -0.125 | -0.4581 | No |
| 83 | CD80 | na | 26418 | -0.126 | -0.4540 | No |
| 84 | CLDN6 | na | 26559 | -0.129 | -0.4526 | No |
| 85 | ITGAV | na | 26724 | -0.133 | -0.4518 | No |
| 86 | CDH3 | na | 27363 | -0.148 | -0.4640 | Yes |
| 87 | ITGB1 | na | 27543 | -0.152 | -0.4627 | Yes |
| 88 | ITGAM | na | 27645 | -0.155 | -0.4591 | Yes |
| 89 | CLDN18 | na | 27780 | -0.159 | -0.4563 | Yes |
| 90 | ITGA6 | na | 27950 | -0.163 | -0.4543 | Yes |
| 91 | CD6 | na | 28024 | -0.165 | -0.4494 | Yes |
| 92 | NFASC | na | 28194 | -0.170 | -0.4472 | Yes |
| 93 | CLDN11 | na | 28639 | -0.183 | -0.4523 | Yes |
| 94 | NCAM2 | na | 28843 | -0.190 | -0.4502 | Yes |
| 95 | ESAM | na | 29009 | -0.195 | -0.4467 | Yes |
| 96 | SIGLEC1 | na | 29736 | -0.217 | -0.4586 | Yes |
| 97 | NRCAM | na | 29816 | -0.219 | -0.4517 | Yes |
| 98 | CNTNAP2 | na | 29838 | -0.219 | -0.4430 | Yes |
| 99 | CD40LG | na | 30278 | -0.236 | -0.4458 | Yes |
| 100 | HLA-DRA | na | 30472 | -0.244 | -0.4411 | Yes |
| 101 | PTPRM | na | 30762 | -0.256 | -0.4387 | Yes |
| 102 | HLA-DPA1 | na | 30909 | -0.263 | -0.4318 | Yes |
| 103 | CDH15 | na | 31165 | -0.276 | -0.4275 | Yes |
| 104 | PVR | na | 31183 | -0.277 | -0.4164 | Yes |
| 105 | CD34 | na | 31199 | -0.278 | -0.4051 | Yes |
| 106 | CD274 | na | 31396 | -0.288 | -0.3986 | Yes |
| 107 | CLDN17 | na | 31516 | -0.295 | -0.3896 | Yes |
| 108 | SPN | na | 31678 | -0.305 | -0.3815 | Yes |
| 109 | CLDN10 | na | 31975 | -0.322 | -0.3765 | Yes |
| 110 | ITGB8 | na | 32130 | -0.330 | -0.3670 | Yes |
| 111 | HLA-DMB | na | 32287 | -0.339 | -0.3573 | Yes |
| 112 | CDH4 | na | 32342 | -0.343 | -0.3444 | Yes |
| 113 | CDH2 | na | 32366 | -0.345 | -0.3305 | Yes |
| 114 | CD226 | na | 32390 | -0.347 | -0.3166 | Yes |
| 115 | CD40 | na | 32420 | -0.349 | -0.3027 | Yes |
| 116 | CLDN14 | na | 32746 | -0.372 | -0.2965 | Yes |
| 117 | ICAM1 | na | 32958 | -0.390 | -0.2862 | Yes |
| 118 | ITGA8 | na | 33651 | -0.470 | -0.2864 | Yes |
| 119 | NRXN3 | na | 33700 | -0.478 | -0.2676 | Yes |
| 120 | NRXN1 | na | 33918 | -0.516 | -0.2522 | Yes |
| 121 | CADM3 | na | 33984 | -0.529 | -0.2318 | Yes |
| 122 | SELL | na | 34027 | -0.539 | -0.2103 | Yes |
| 123 | NCAM1 | na | 34105 | -0.558 | -0.1890 | Yes |
| 124 | HLA-DPB1 | na | 34158 | -0.570 | -0.1665 | Yes |
| 125 | MADCAM1 | na | 34188 | -0.578 | -0.1431 | Yes |
| 126 | JAM2 | na | 34268 | -0.600 | -0.1201 | Yes |
| 127 | CLDN2 | na | 34525 | -0.714 | -0.0974 | Yes |
| 128 | HLA-DQA2 | na | 34566 | -0.745 | -0.0672 | Yes |
| 129 | HLA-DQA1 | na | 34604 | -0.798 | -0.0347 | Yes |
| 130 | JAM3 | na | 34668 | -0.909 | 0.0017 | Yes |
Table: GSEA details [plain text format]

  

Fig 2: KEGG\_CELL\_ADHESION\_MOLECULES\_CAMS      
 Blue-Pink O' Gram in the Space of the Analyzed GeneSet

  

Fig 3: KEGG\_CELL\_ADHESION\_MOLECULES\_CAMS: Random ES distribution      
 Gene set null distribution of ES for **KEGG\_CELL\_ADHESION\_MOLECULES\_CAMS**
